# *Saccorhiza polyschides*—A Source of Natural Active Ingredients for Greener Skincare Formulations

**DOI:** 10.3390/molecules27196496

**Published:** 2022-10-01

**Authors:** Patrícia Susano, Joana Silva, Celso Alves, Alice Martins, Susete Pinteus, Helena Gaspar, Márcia Inês Goettert, Rui Pedrosa

**Affiliations:** 1 MARE-Marine and Environmental Sciences Centre/ARNET-Aquatic Research Network, Polytechnic of Leiria, 2520-630 Peniche, Portugal; 2 BioISI-Biosystems and Integrative Sciences Institute, Faculty of Sciences, University of Lisbon, 1749-016 Lisboa, Portugal; 3 Cell Culture Laboratory, Postgraduate Programme in Biotechnology, University of Vale do Taquari-Univates, Lajeado 95914-014, RS, Brazil; 4 Department of Pharmaceutical and Medicinal Chemistry, Institute of Pharmacy, Eberhard Karls Universität Tübingen, D 72076 Tübingen, Germany; 5 MARE/ARNET, ESTM, Polytechnic of Leiria, 2520-614 Peniche, Portugal

**Keywords:** bioactive compounds, brown macroalgae, dermatological potential, oxidative stress, anti-inflammatory activity

## Abstract

The growing knowledge about the harmful effects caused by some synthetic ingredients present in skincare products has led to an extensive search for natural bioactives. Thus, this study aimed to investigate the dermatological potential of five fractions (F1–F5), obtained by a sequential extraction procedure, from the brown seaweed *Saccorhiza polyschides*. The antioxidant (DPPH, FRAP, ORAC and TPC), anti-enzymatic (collagenase, elastase, hyaluronidase and tyrosinase), antimicrobial (*Staphylococcus epidermidis*, *Cutibacterium acnes* and *Malassezia furfur*), anti-inflammatory (nitric oxide, tumor necrosis factor-α, interleukin-6 and interleukin-10) and photoprotective (reactive oxygen species) properties of all fractions were evaluated. The ethyl acetate fraction (F3) displayed the highest antioxidant and photoprotective capacity, reducing ROS levels in UVA/B-exposed 3T3 fibroblasts, and the highest anti-enzymatic capacity against tyrosinase (IC_50_ value: 89.1 µg/mL). The solid water-insoluble fraction (F5) revealed the greatest antimicrobial activity against *C. acnes* growth (IC_50_ value: 12.4 µg/mL). Furthermore, all fractions demonstrated anti-inflammatory potential, reducing TNF-α and IL-6 levels in RAW 264.7 macrophages induced with lipopolysaccharides. Chemical analysis of the *S. polyschides* fractions by NMR revealed the presence of different classes of compounds, including lipids, polyphenols and sugars. The results highlight the potential of *S. polyschides* to be incorporated into new nature-based skincare products.

## 1. Introduction

Skin is the largest organ of the human body, and it is made up of three different layers, which are as follows: (i) the epidermis, the outermost layer constituted by keratinocytes; (ii) dermis, formed by fibroblasts; and (iii) hypodermis, consisting mostly of connective tissue and fat. As the first barrier to the external environmental, skin plays a key role in protecting against external insults (e.g., ultraviolet radiation (UVR) exposure, pollution and microorganisms), and in maintaining internal homeostasis [1].

One of the major skin problems is premature skin aging, characterized by the progressive loss of structural and functional cell integrity [2]. This process can occur due to endogenous factors (intrinsic aging) or exogenous factors (photoaging). Intrinsic aging can be caused by changes in the skin over time related with metabolism, hormones, and genetic predisposition, while photoaging is related to external insults, mainly exposure to UVR [2]. Its exposure to these stressors triggers a sequence of biological events, such as reactive oxygen species (ROS) production. ROS are naturally produced by organisms and the human body produces antioxidants as a defense mechanism to eliminate them. However, when there is an imbalance between the production of ROS and the defensive system’s ability to neutralize these radicals, an oxidative stress condition is generated [3], which can cause dermatological disorders and consequently, skin aging.

Free radicals formed due to oxidative stress play an important role in skin aging (intrinsic and photoaging) [4]. The oxidative stress causes an increase in ROS levels, which can induce the expression of matrix metalloproteinases (MMPs) [5] and up-regulate the mitogen-activated protein kinases (MAPKs) pathway, increasing the overexpression of activator protein 1 (AP-1), leading to transcript factor activation and, consequently, increase in inflammatory cytokines production [6]. The increased expression of MMPs, such as collagenase (MMP-1), gelatinases (MMP-2 and MMP-9) and elastase (MMP-12), triggers the degradation of extracellular matrix proteins, namely collagen and elastin [7]. Besides collagen and elastin, hyaluronic acid (HA) is a major component of the extracellular matrix, being responsible for skin repair, hydration and flexibility maintenance [8]. As a result, decreased levels of collagen, elastin, and HA compromise the function and repair capacity of the skin, turning it thinner, fragile, and unable to maintain natural elasticity and hydration, leading to the development of visible signs of skin aging. One of the approaches to minimize these phenomena is to inhibit the enzymes involved in these molecules’ degradation [9]. Additionally, melanin, a major component of the skin, hair, and eye color, is synthesized by melanogenesis through tyrosinases [10]. The excessive and unprotected exposure to UVR promotes an over production of melanin, leading to several hyperpigmentation disorders, including melasma, age spots and blemishes, resulting in premature aging appearance. In this sense, the inhibition of tyrosinases is considered an approach for the treatment of the hyperpigmentation and can be used as a whitening agent of the skin [11]. Besides the role of the above-described enzymes in cutaneous aging, inflammation can also compromise skin homeostasis. Effectively, an increase in ROS levels activates NF- κB and MAPKs signaling pathways, leading to the release of inflammatory metabolites, such as nitric oxide (NO), and transcription of pro-inflammatory mediators, namely interleukins (IL) (e.g., IL-1, IL-6 and IL-8) and tumor necrosis factor-α (TNF- α). The release of the pro-inflammatory mediators has been associated with numerous disorders and pathologies that are also associated with the development and progression of skin diseases, such as acne, atopic dermatitis, melanoma, or cancer [12]. In addition to oxidative stress, enzymatic degradation and inflammatory conditions, several microorganisms also have an important role in the cutaneous equilibrium. Healthy skin is composed of various commensal microorganisms, such as *Staphylococcus epidermidis*, *Cutibacterium acnes* and *Malassezia furfur* that belong to the skin’s microbiome. These play a major role in protecting the human body against external pathogens, such as bacteria and viruses. However, in an altered microbial state, these microorganisms can become opportunistic, leading to the development of infections and dermatitis. Thus, it is important to find bioactive components that contribute to the maintenance of a balanced skin microbiome [13].

One of the primary focuses of the skincare industry is the search for new natural ingredients with a range of bioactivities, such as antioxidant, anti-aging, anti-inflammatory, among others, in order to repair and maintain the health of the skin, protecting it from the harmful effects of oxidative stress, as well as in the treatment of inflammatory events [1]. However, although there are many products that protect the skin against external aggressions, most of them have in their constitution chemical compounds that are associated with phototoxicity, cellular mutation and low stability. For instance, butylhydroxytoluene (BHT) is widely used in cosmetic formulations as an antioxidant, maintaining the properties and performance of the products exposed to air; however, different studies have reported its possible carcinogenic and harmful effects on health [14]. Thus, the search for new skincare products is crucial and it is important to find alternatives of natural origin. In this sense, seaweeds can be the key for the development of new natural skincare formulations as they have revealed antioxidant, anti-aging, photoprotective, antimicrobial and anti-inflammatory properties [15,16,17,18,19].

*Saccorhiza polyschides* (Lightfoot) (Batters 1902) is a brown seaweed (Phaeophyceae) with an abundant presence in the lower littoral zone of the Northeast Atlantic Coast, where it colonizes hard substrata in the sublittoral [20]. *Saccorhiza polyschides* has been the subject of previous studies and has revealed antioxidant, neuroprotective and biofertilizer potential [21,22,23,24]. Therefore, this study aimed to evaluate the dermatological potential of *Saccorhiza polyschides*, evaluating its antioxidant, anti-aging, antimicrobial, anti-inflammatory and photoprotective capacities. For this purpose, *S. polyschides* was subjected to a sequential extraction with solvents allowed in the cosmetic industry. Five fractions of different polarities (F1–F5) were obtained, and their chemical profiles were evaluated by proton NMR spectroscopy.

## 2. Results

**Figure 1:**
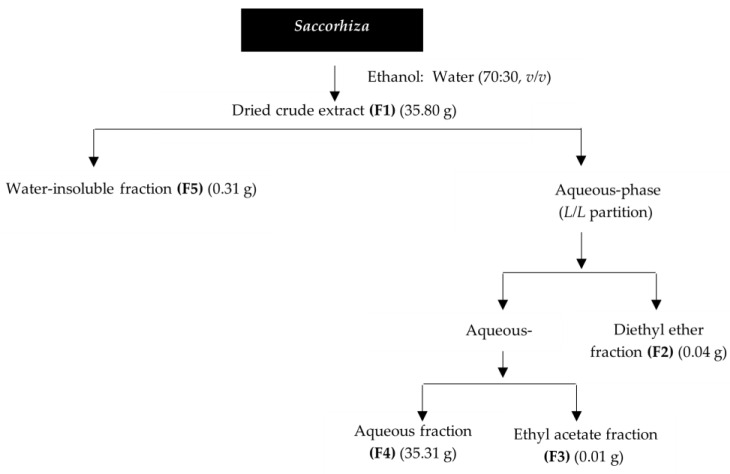
*Saccorhiza polyschides* extraction flowchart.

The dermocosmetic potential of *S. polyschides* was evaluated through a set of *in vitro* assays. Results of the antioxidant, photoprotective, anti-enzymatic, antimicrobial and anti-inflammatory activities of different fractions from this seaweed are presented, together with a preliminary chemical screening of each fraction. These fractions were obtained as follows: seaweed powdered samples (180.00 g) were extracted overnight at room temperature with ethanol: water (70:30, *v*/*v*), under constant stirring. The resulting liquid extract was evaporated until dryness to obtain the crude extract (F1). Then, this fraction was re-suspended in hot (70 °C) water, cooled, and filtered through filter paper, producing a solid insoluble fraction retained in the filter (F5), and a liquid aqueous phase. This last one was subjected to a *L*/*L* partition, firstly with diethyl ether and then with ethyl acetate. Organic phases were concentrated to dryness, resulting in the diethyl ether (F2), ethyl acetate (F3) and aqueous (F4) fractions. All fractions were concentrated under reduced pressure, at low temperature (40 °C), while the remaining F4 was freeze-dried. A flowchart of the *S. polyschides* extraction process is illustrated in Figure 1. 

### 2.1. Antioxidant Capacity

The antioxidant potential of the *S. polyschides* fractions was evaluated through the 2,2-diphenyl-1-picrylhydrazyl (DPPH) radical scavenging activity, ferric reducing antioxidant power (FRAP), and oxygen radical absorbance capacity (ORAC) assays and results associated with the total phenolic content (TPC), using butylhydroxytoluene (BHT) as a reference compound (Table 1).

The ethyl acetate fraction (F3) displayed the highest phenolic content (199.9 ± 23.7 mg PE/g). Furthermore, this fraction and the diethyl ether fraction (F2) revealed a strong capacity to scavenge oxygen reactive species, demonstrating the greatest values of ORAC (3126.0 ± 225.1 and 1715.8 ± 44.6 µmol TE/g, respectively). These values were much higher than those obtained with the antioxidant standard BHT (142.9 ± 9.1 µmol TE/g). F2 and F3 also showed ability to reduce Fe (III) ions (1081.6 ± 33.3 and 828.2 ± 21.2 µM FeSO_4_/g, respectively). On the other hand, none of the fractions presented a relevant DPPH radical scavenging activity.

### 2.2. Anti-Enzymatic Activity

**Figure 2:**
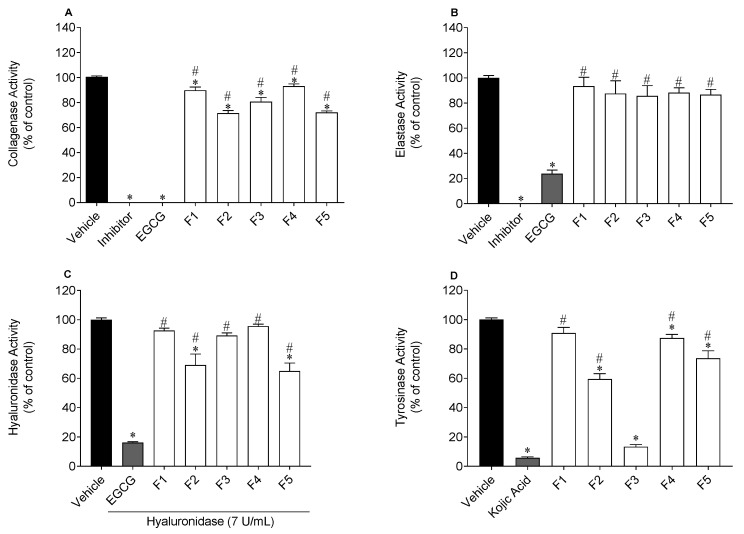
Enzymatic inhibitory activity of *Saccorhiza polyschides* fractions (200 µg/mL) on collagenase (**A**), elastase (**B**), hyaluronidase (**C**) and tyrosinase (**D**). Epigallocatechin gallate (EGCG) was used as the positive control of collagenase, elastase and hyaluronidase. Kojic acid was used as the positive control of tyrosinase. Symbols represent significant differences (one-way ANOVA, Dunnett’s test; *p <* 0.05) when compared to vehicle (*) and EGCG or kojic acid (#).

**Figure 3:**
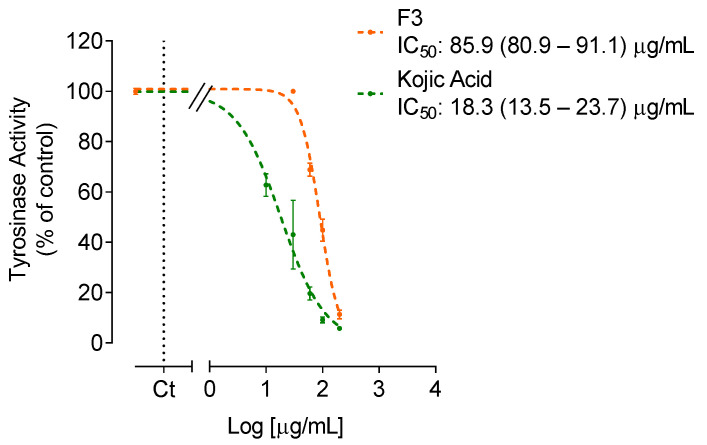
Dose-response curves (10–200 µg/mL) for enzymatic inhibitory activities of *Saccorhiza polyschides* fraction (F3) and kojic acid on tyrosinase, expressed as IC_50_ value.

The inhibitory effects of *S. polyschides* fractions on collagenase, hyaluronidase, elastase and tyrosinase activities are shown in Figure 2 and Figure 3.

For fraction F3 that inhibited tyrosinase activity by more than 50%, its IC_50_ value was determined, and the dose-response curves are shown in Figure 3.

The *S. polyschides* fractions, mostly F2, F3 and F5, demonstrated inhibitory effects against the activity of collagenase, hyaluronidase and tyrosinase. Regarding collagenase, fractions F2 and F5 revealed the greatest potential, reducing the activity of this enzyme approximately by 29% and 28%, respectively. However, these fractions showed a lower activity than the reference standard EGCG. Nonetheless, all the fractions demonstrated capacity to reduce collagenase activity, unlike elastase, since none of the fractions reduced its activity. Regarding hyaluronidase, fractions F2 and F5 significantly reduced its activity by approximately 31% and 35%, respectively. Finally, concerning tyrosinase, fraction F3 revealed the strongest inhibitory capacity of tyrosinase activity (IC_50_ value of 85.9 µg/mL), but with lower activity than the positive control kojic acid (IC_50_ value of 18.3 µg/mL), (Figure 3). The fraction F2 reduced the activity of this enzyme by approximately 41% (Figure 2).

### 2.3. Antimicrobial Activity

**Figure 4:**
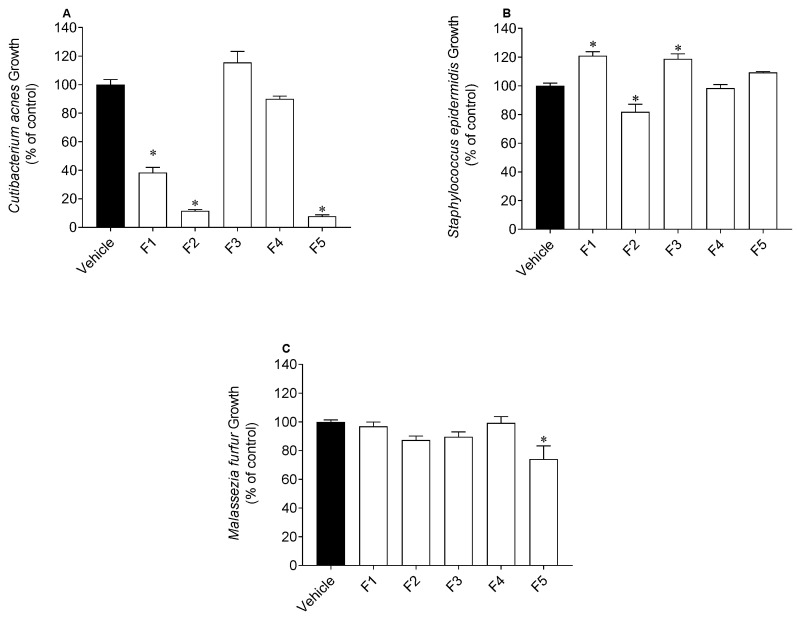
Effect of *Saccorhiza polyschides* fractions (200 µg/mL) on the growth of *Cutibacterium acnes* (**A**)*, Staphylococcus epidermidis* (**B**) and *Malassezia furfur* (**C**). Symbol (*) represents significant differences (one-way ANOVA, Dunnett’s test; *p <* 0.05) when compared to vehicle.

The effects of *S. polyschides* fractions on the growth of *S. epidermidis*, *C. acnes* and *M. furfur* are presented in Figure 4.

For the samples that decreased the microbial growth by more than 50%, their IC_50_ values were determined (Table 2). As presented in Figure 4, the *S. polyschides* fractions affected the growth of microorganisms, specifically *C. acnes*. The water-insoluble fraction (F5) revealed the highest inhibitory potential against *C. acnes* (IC_50_ value of 12.4 µg/mL), followed by F2 (IC_50_ value of 33.4 µg/mL) and F1 (IC_50_ value of 39.0 µg/mL) (Table 2). However, these fractions showed lower activity than the reference drug, oxytetracycline (IC_50_ value of 0.07 µg/mL). Concerning *S. epidermidis*, F2 revealed the greatest inhibitory potential, reducing its growth by approximately 20%. F5 was the only fraction with inhibitory activity against *M. furfur*, reducing its growth by, approximately, 15% (Figure 4). Although F2 and F5 reduced the growth of *S. epidermidis* and *M. furfur*, respectively, they demonstrated lower activity than the reference drug (IC_50_ value of 12.4 and 11.4 µg/mL, respectively).

### 2.4. Nitric Oxide Levels Produced by RAW 264.7 Macrophages

**Figure 5:**
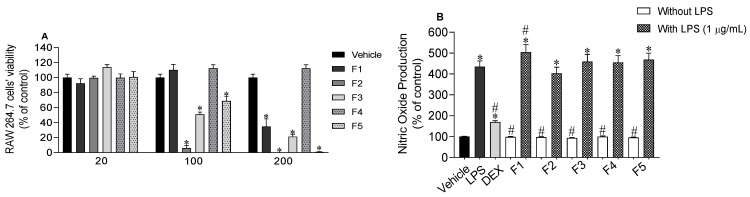
Effects of *Saccorhiza polyschides* fractions (20–200 µg/mL) on RAW 264.7 cell’s viability following treatment for 24 h. (**A**) Nitric oxide (NO) levels (% of control) following exposure with *Saccorhiza polyschides* fractions (20 µg/mL), LPS (1 µg/mL), dexamethasone (DEX) (20 µg/mL) and LPS in the presence of *Saccorhiza polyschides* fractions (20 µg/mL) (**B**). Symbols represent significant differences (one-way ANOVA, Dunnett’s test; *p <* 0.05) when compared to vehicle (*) and LPS (#).

Firstly, the viability of RAW 264.7 cells when exposed to *S. polyschides* fractions was evaluated (Figure 5A). At 20 µg/mL, none of the fractions exhibited cytotoxicity and so, this concentration was selected to quantify nitric oxide (NO) production by RAW 264.7 cells in normal and inflammatory conditions. None of the *S. polyschides* fractions stimulated NO production when exposed to cells. On the other hand, none of these fractions decreased the NO levels stimulated by LPS treatment (Figure 5B). However, fraction F1 significantly increased the NO levels promoted by LPS treatment (Figure 5B).

### 2.5. Inflammatory and Anti-Inflammatory Cytokines Levels

**Figure 6:**
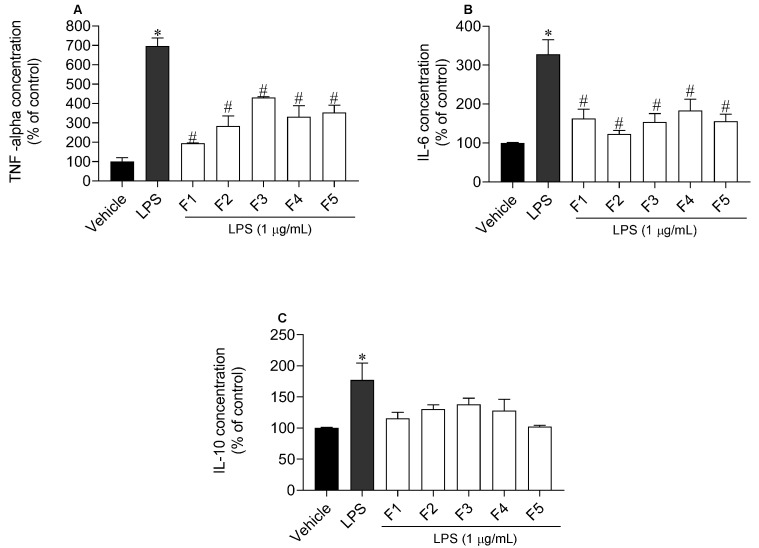
Concentration levels of TNF-α (**A**), IL-6 (**B**) and IL-10 (**C**) cytokines on RAW 264.7 cells treated with LPS (1 µg/mL) in the presence of *Saccorhiza polyschides* fractions (20 µg/mL). Symbols represent significant differences (one-way ANOVA, Dunnett’s test; *p <* 0.05) when compared to vehicle (*) and LPS (#).

The release of two pro-inflammatory cytokines, namely TNF-α and IL-6, and of the anti-inflammatory cytokine IL-10, by RAW 264.7 cells treated with LPS (1 µg/mL) in the presence/absence of *S. polyschides* fractions (20 µg/mL) was studied (Figure 6). Concerning TNF-α, all fractions reduced its levels, although F1, followed by F2, showed the highest effect. When inflammation was induced by LPS, TNF-α release increased to 696.6 ± 40.2% compared with LPS treatment; however, when the cells were treated with F1 and F2, TNF-α levels decreased to 194.2 ± 1.9% and 283.9 ± 45.2%, respectively (Figure 6A). Regarding IL-6, all fractions decreased the release of this cytokine, but F2 exhibited the greatest potential, decreasing its levels (% of control) from 327.5 ± 34.4% to 123.2 ± 7.7% (Figure 6B). Lastly, none of these fractions stimulated the production of the anti-inflammatory cytokine IL-10 (Figure 6C).

### 2.6. Photoprotective Capacity

**Figure 7:**
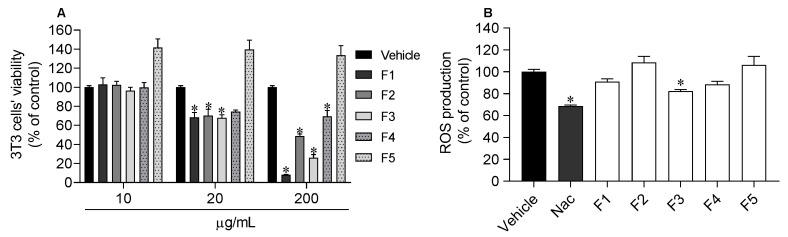
Effects of *Saccorhiza polyschides* fractions (10–200 µg/mL) on 3T3 cell’s viability after 24 h of treatment. (**A**) Reactive oxygen species (ROS) levels (% of control) on 3T3 fibroblasts following exposure to UV radiation (12.5 mJ/cm^2^; 1 h), in the presence/absence of *Saccorhiza polyschides* fractions (10 µg/mL) and *N*-acetyl-l-cysteine (NAC, 10 µg/mL) (**B**). Symbols (*) represent significant differences (one-way ANOVA, Dunnett’s test; *p <* 0.05) when compared to vehicle.

The viability of 3T3 fibroblasts was evaluated following the treatment with *S. polyschides* fractions (Figure 7A).

At 10 µg/mL, none of the fractions showed cytotoxicity and so, this concentration was selected for the photoprotective assay. The ethyl acetate fraction (F3) was the only fraction that exhibited photoprotective ability, reducing the ROS production (82.3 ± 1.5%) induced by UV exposure to values close to those of the standard (NAC) (68.6 ± 0.9%) (Figure 7B).

### 2.7. NMR Chemical Profile

**Figure 8:**
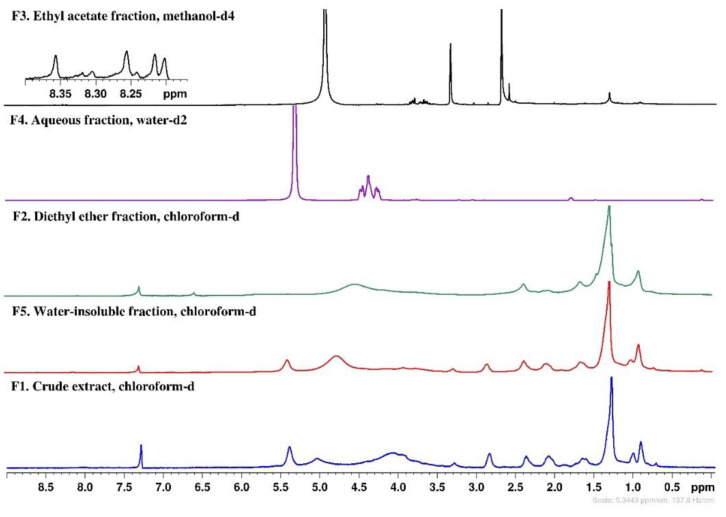
^1^H NMR spectra (400 MHz) of *Saccorhiza polyschides* fractions.

The chemical profile of the *S. polyschides* fractions (F1–F5) was evaluated by ^1^H NMR and the corresponding spectra are depicted in Figure 8.

Some similarities were observed in the ^1^H NMR spectra of the crude extract (F1) and the water-insoluble fraction (F5). Both samples evidenced signals in the region of 0.91–2.86 ppm that could be caused by lipophilic compounds, such as fatty acids, sterols, terpenes, and other lipids [25,26,27]. Proton signals of pigments, probably fucoxanthin [28], were also observed in both fractions. Additionally, an intense peak at 2.36 ppm may suggest the presence of protons bound to carbon atoms in the alpha position to unsaturated groups in allylic, carbonyl, or amino groups [26]. The main difference between both fractions relates to the presence of signals (3.85–4.35 ppm) most probably from sugars, including polysaccharides, in the crude extract. Progressing to the enriched fractions, as a result of liquid–liquid partitions, spectra of less complexity can be observed. In particular, the diethyl ether fraction (F2) also evidences signals (0.91–2.36 ppm) probably from the less polar compounds, while the aqueous fraction (F4) clearly shows intense signals in the range of 3.77–4.01 ppm, which can be attributed to mannitol, a common polyol identified in many brown seaweeds [26]. Concerning the ethyl acetate fraction (F3), in expanded spectra, it was possible to observe signals in the range of 8.17–8.40 ppm, which can be attributed to the aromatic protons of phenolic compounds, probably phlorotannins, a group of phenolics very common in brown seaweeds [29,30].

## 3. Discussion

Due to the population’s growing awareness of the skin’s importance for body homeostasis, there is significant demand for natural and effective skincare products that delay skin aging and improve its repair capacity. In recent years, seaweeds have been revealed as a source of natural bioactive compounds (e.g., pigments, sulfated polysaccharides and phenolic compounds) [31], which can be an alternative for the development of new skin formulations [32,33].

Exposure to UV radiation is responsible for causing several types of damage to the skin, including photoaging, resulting in ROS-mediated secondary reactions [34]. In the present work, the different fractions obtained from *S. polyschides* were evaluated for their antioxidant capacity using three complementary methods (DPPH, FRAP and ORAC) and results related to the total phenolic content (TPC). The diethyl ether (F2) and ethyl acetate (F3) fractions showed the highest antioxidant capacity. These fractions revealed a strong ability to scavenge reactive oxygen species, demonstrating the highest values of ORAC (1715.8 ± 44.6 and 3126.0 ± 225.1 µmol TE/g, respectively), and ability to reduce Fe (III) ions (1081.6 ± 33.3 and 828.2 ± 21.2 µM FeSO_4_/g, respectively). Fraction F2, and particularly F3, displayed the highest phenolic content, which was supported by the signals in the aromatic region (8.1–8.4 ppm) of their NMR spectra, which suggest the presence of phlorotannins [30]. These secondary metabolites are known for their strong biological activities, especially antioxidant activity [35]. Pinteus et al. [21] also studied the antioxidant potential of *S. polyschides* collected off the Peniche coast, obtaining a lower phenolic content (66.89 ± 0.002 mg PE/g) and a lower value of ORAC (237.61 ± 3.13 µmol TE/g), when compared to F2 and F3. However, the same study reports that *S. polyschides* had the greatest capacity for scavenging DPPH radicals (EC_50_ value: 49.2 µg/mL), while in the present study, none of the fractions revealed a capacity to reduce these radicals. These differences in the antioxidant potential of *S. polyschides* may be related to the solvents and the extraction methodology performed in the two studies. Lim and co-workers [36] evaluated the phenolic content of the seaweed *Sargassum serratifolium* using solvents with distinct characteristics. The data displayed that, depending on the solvent used, the phenolic content differs, with the ethyl acetate fraction being the fraction with the highest phenolic content (105.0 ± 2.4 mg PE/g), corroborating our results.

With aging, components of the skin’s extracellular matrix (e.g., collagen, elastin, HA) tend to decrease, leading to a weakening of the skin structure [37]. Furthermore, excessive exposure to UV radiation leads to ROS overproduction, which can result in pigmentation disorders. Herein, the anti-enzymatic activities of *S. polyschides* fractions on collagenase, elastase, hyaluronidase and tyrosinase were studied. The results demonstrated inhibitory effects against the activity of collagenase, hyaluronidase, and tyrosinase, being more pronounced in the last enzyme. F3 showed strong inhibitory activity of tyrosinase (IC_50_ value: 89.1 µg/mL), which may be related to its high content in phenolic compounds, since this group of molecules has been reported for their anti-enzymatic capacity [33,38]. Barbosa and co-workers [39] observed that phlorotannins extracted from *Fucus* species inhibited the activity of tyrosinase, revealing a correlation between the total content of phlorotannins and the potential to inhibit the activity of this enzyme. Freitas et al. [40] evaluated the anti-enzymatic activity of *Fucus spiralis* and observed that the phlorotannin-enriched fraction had the greatest inhibitory effect against collagenase and elastase activities.

A balanced skin microbiome is crucial to maintain healthy skin. The microbiome is composed of microorganisms from different species, which live in symbiosis and provide defense against external pathogens. However, when the skin’s microbiome is unbalanced, it can lead to the development of several skin disorders, such as acne, dermatitis, rosacea, etc. [41]. In this study, the three microorganisms studied are members of the human skin microbiome. The results showed that the crude extract (F1), the diethyl ether (F2) and the water insoluble (F5) fractions of *S. polyschides* decreased the growth of *C. acnes* (IC_50_ value of 39.0, 33.4 and 12.4 µg/mL, respectively). As evidenced by ^1^H NMR spectra, these fractions are very rich in lipophilic compounds, and they have been reported for their high antimicrobial activity [42,43]. In a study performed by Rabah et al. [44], the antimicrobial activity of lipophilic fractions from *Allium triquetrum* showed capacity to inhibit *Staphylococcus aureus* growth. Regarding *S. epidermidis,* fraction F2 decreased the growth of this microorganism, while fractions F1 and F3 stimulated it. Recent studies have shown that *S. epidermidis* and *C. acnes* interact together and are critical for skin homeostasis regulation. Stimulating the growth of *S. epidermidis* may be beneficial in the acne ‘pathophysiology, as this pathology causes an increase in *C. acnes* strains and a decrease in *S. epidermidis* [45]. In this sense, the exploitation of fraction F1 can be a good approach for the treatment of acne, since it has shown the ability to stimulate the growth of *S. epidermidis* and to reduce the growth of *C. acnes*, allowing restoration of the natural balance of skin microbiota. Concerning *M. furfur*, only F5 showed capacity to inhibit the growth of this microorganism, which can be related to the high resistance of yeast strains to antimicrobial agents, since yeasts present an additional barrier to defend themselves against chemical compounds [46].

Inflammation is associated with the development and progression of skin diseases [12] and is crucial to develop more effective therapeutic strategies, with fewer side effects for patients [47]. Herein, the ability of *S. polyschides* fractions to inhibit the production of NO and inflammatory cytokines in murine macrophage cells (RAW 264.7) treated with LPS was evaluated. At the non-toxic concentration (20 µg/mL), none of the *S. polyschides* fractions induced an inflammatory condition, since none of them induced the production of NO. On the other hand, none of the fractions decreased the NO levels induced by LPS treatment. Besides the quantification of NO levels, the potential of *S. polyschides* fractions to inhibit the release of TNF-α, IL-6 and IL-10 cytokines was also evaluated.

In an inflammatory process, TNF-α is initially released, leading to an increased expression of other cytokines and, at the same time, IL-6 stimulates the release of other inflammatory mediators, triggered by elevated ROS production [48]. In addition, these cytokines can stimulate p-38α MAPK, which participates in the regulation of inflammatory responses [49]. On the other hand, IL-10 plays an important role in the immune system, by acting as an anti-inflammatory mediator in the subsequent level of the inflammatory cascade [17].

In the present work, the levels of TNF-α, IL-6 and IL-10 were enhanced with the treatment with LPS, when compared to the vehicle, which is indicative of an inflammation status. In general, all fractions significantly reduced the TNF-α and IL-6 levels. On the other hand, none of the fractions were able to stimulate the production of IL-10. These results agree with those found in the literature [50,51], where brown seaweeds also revealed anti-inflammatory potential. Han and co-workers [52] evaluated the anti-inflammatory effect of sargacromenol isolated from *Sargassum horneri* in RAW 264.7 macrophages stimulated by LPS. Sargacromenol, besides reducing the production of NO and ROS, also decreased the mRNA expression of inflammatory cytokines, namely IL-1β, IL-6 and TNF-α, and inflammatory mediators, namely iNOS and COX-2. A study conducted by De la Fuente et al. [53] revealed the anti-inflammatory potential of extracts from the brown seaweed *Cystoseira amentacea* that inhibit the production of NO and the expression of IL-1α, IL-6 and COX-2 genes, suggesting their use in anti-aging formulations and cosmetic lotions for inflamed and/or damaged skin. Park and co-workers [54] revealed the anti-inflammatory effects of fucoidan, through inhibition of NF-κB, MAPK and Akt activation in lipopolysaccharide-induced BV2 microglia cells, suggesting that the anti-inflammatory effects were also due to the inhibition of the MAPK pathway.

The skin naturally has antioxidant mechanisms capable of blocking ROS, preventing cellular destabilization. However, although effective, this defense system is usually overloaded due to excessive UVR exposure [55]. In this work, the photoprotective potential of *S. polyschides* fractions against UVA and UVB radiation was studied in fibroblast cells (3T3), and it was verified that at the non-toxic concentration (10 µg/mL), only the F3 fraction protected the cells from ROS resulting from UVR exposure. In fact, the phenolic compounds present in brown seaweeds have been described for their photoprotective capacity, as they can absorb UVR [56]. In a study conducted by Yu and co-workers [57], a polyphenolic rich extract from the brown seaweed *Sargassum muticum* showed protective effects against ROS damage in HaCaT cells irradiated with UVB, while Prasedya et al. [58] revealed the potential against UVA radiation of ethanolic extracts of *Sargassum cristafolium.*

## 4. Materials and Methods

### 4.1. Saccorhiza Polyschides Collection and Preparation

The brown seaweed *S. polyschides* was collected at Portinho da Areia Sul beach, Peniche, Portugal (39°21′13.2″ N 9°23′19.3″ W) and transported to the laboratory. It was identified by the marine biologist Dr. Susete Pinteus, using an identification guide cross-checked with AlgaeBase (htttps://www.algaebase.org (accessed on 28 June 2022)). A specimen standard (SP052021) was deposited in our lab. After cleaned and washed with seawater to remove invertebrate organisms, epiphytes and debris, *S. polyschides* was frozen at −20 °C and freeze-dried (Scanvac Cool Safe, LaboGene, Lynge, Denmark). The dried algal material was ground into a powder in a grinder, and stored at room temperature protected from light, until the extraction procedures.

### 4.2. Saccorhiza Polyschides Extraction

Freeze-dried biomass of *S. polyschides* (180.00 g) was extracted sequentially, as previously reported in the Results section.

The following five fractions were obtained: the total hydroalcoholic extract (F1), the diethyl ether (F2), ethyl acetate (F3), aqueous (F4), and the solid water insoluble (F5) fractions.

Solvents (*p.a*) used in the extraction and fractionation processes were supplied by VWR-BDH Chemicals-Prolabo (Leuven, Belgium), while pure water was obtained in a MilliQ system (Advantage A10 Milli-Q lab, Merck, Darmstadt, Germany).

### 4.3. Biological Activities of Saccorhiza Polyschides

For *in vitro* bioassays, a stock solution of each fraction (F1–F5) from *S. polyschides* was prepared in dimethyl sulfoxide (DMSO) at a concentration of 20 mg/mL.

#### 4.3.1. Antioxidant Capacity

The antioxidant potential of *S. polyschides* fractions was evaluated through three different methods, namely 2,2-diphenyl-1-picrylhydrazyl (DPPH) radical scavenging activity, ferric reducing antioxidant power (FRAP), and oxygen radical absorbance capacity (ORAC), and results associated with the total phenolic content (TPC) [18].

##### 2,2-Diphenyl-1-Picrylhydrazyl (DDPH) Radical Scavenging Activity

The capacity of *S. polyschides* fractions (200 µg/mL) to scavenge the DPPH radical was performed according to the work of Brand-Williams and co-workers [59]. The reaction occurred for 30 min in the dark, and absorbance was measured at 517 nm in a microplate reader (Epoch Microplate Reader, BioTek^®^ Instruments, Winooski, VT, USA).

##### Ferric Reducing Antioxidant Power (FRAP)

The FRAP assay was performed as described by Benzie and Strain [60], adapted to microscale with slight modifications [18]. FRAP reagent was prepared with 0.3 M acetate buffer (pH 3.6), 10 mM of 2,4,6-Tris (2-pyridyl)-s-triazine (TPTZ) in 40 mM HCl and 20 mM ferric solution, using FeCl_3_ at a ratio of 10:1:1 and incubated at 37 °C. *S. polyschides* fractions were added to FRAP reagent and incubated in the dark for 30 min, at 37 °C, and the absorbance was measured at 593 nm using a microplate reader (Epoch Microplate Reader, BioTek^®^ Instruments, Winooski, VT, USA). FeSO_4_ was used as the standard for the calibration curve, and the results were expressed as micromolar of FeSO_4_ equivalents per gram of dry extract (µM of FeSO_4_/g of extract).

##### Oxygen Radical Absorbance Capacity (ORAC)

The ORAC assay was performed according to the work of Dávalos and co-workers [61]. Seaweed fractions were pre-incubated with fluorescein (70 nM) for 15 min at 37 °C. After this time, 2,2′-azobis (2-methylpropionamidine) dihydrochloride (AAPH) solution (12 mM) was added and the fluorescence (λ excitation: 458 nm; λ emission: 520 nm) was recorded every minute for 240 min using the microplate reader (Multimodal Synergy H1, BioTek^®^ Instruments, Winooski, VT, USA). Trolox was used as the standard antioxidant, and the results were expressed as micromoles of Trolox equivalents per gram of dry extract (µmol TE/g of extract).

##### Quantification of Total Phenolic Content (TPC)

TPC was determined by the Folin-Ciocalteu method [62], with slight modifications [18]. This method is based on the colorimetric reaction of phenolic substances with Folin-Ciocalteu reagent. After 1 h of reaction in the dark, the absorbance was measured at 750 nm using a microplate reader (Epoch Microplate Reader, BioTek^®^ Instruments, Winooski, VT, USA). Phloroglucinol was used as the standard for the calibration curve, and TPC was expressed in milligrams of phloroglucinol equivalents per gram of dry extract (mg PE/g of extract).

#### 4.3.2. Enzymatic Inhibitory Activity

The inhibitory effects of *S. polyschides* fractions on collagenase (type IV), elastase, hyaluronidase, and tyrosinase enzymes were evaluated as described in the following sections.

##### Anti-Collagenase Activity

The anti-collagenase activity was determined using the EnzChek™ Gelatinase/Collagenase Assay Kit (# E12055, Invitrogen™, ThermoFisher Scientific, Waltham, MA, USA). Epigallocatechin gallate (EGCG) (200 µg/mL) was used as the positive control and the results were expressed as arbitrary fluorescence units per minute (∆ fluorescence (a.u.)/min) as a percentage of the control.

##### Anti-Elastase Activity

The anti-elastase activity was determined using the EnzChek™ Elastase Assay Kit (# E12056, Invitrogen™, ThermoFisher Scientific, Waltham, MA, USA). EGCG (200 µg/mL) was used as the positive control and the results were expressed as arbitrary fluorescence units per minute (∆ fluorescence (a.u.)/min) as a percentage of the control.

##### Anti-Hyaluronidase Activity

The anti-hyaluronidase activity was determined following the method described by Yahaya and Don [63], with slight modifications and adapted to the microscale [40]. Briefly, 3 µL of each fraction was mixed with 5 µL of hyaluronidase (7 U/mL) and 67 µL of enzyme diluent (20 mM sodium phosphate, 77 mM sodium chloride and 0.01% bovine serum albumin (BSA); pH 7.0 at 37 °C) and pre-incubated at 37 °C for 10 min. After that, 25 µL of hyaluronic acid solution (0.03% in 300 mM sodium phosphate; pH 5.35 at 37 °C) was added and incubated for 45 min at 37 °C. Hyaluronic acid was then precipitated using 200 µL of acidic albumin solution (24 mM sodium acetate, 79 mM acetic acid and 0.1% BSA; pH 3.75 at 25 °C). After 10 min at room temperature, the absorbance was measured at 600 nm. The absorbance in the absence of enzyme was used as the control value for maximum inhibition. The hyaluronidase inhibitory activity of each fraction was determined as follows: ((Abssample-Abssample blank)/Abscontrol), where Abssample is the absorbance of the sample with hyaluronidase, hyaluronic acid, and acidic albumin, Abssample blank is the absorbance of the sample, hyaluronidase, and acidic albumin, and Abscontrol is the absorbance of the hyaluronic acid and acidic albumin (without hyaluronidase).

##### Anti-Tyrosinase Activity

The inhibition of tyrosinase activity was performed as described by Senol and co-workers [64], with slight modifications [18]. This method is based on the oxidation of L-3,4-dihydroxyphenylalanine (L-DOPA) by tyrosinase. Briefly, 2 µL of each fraction was mixed with 68 µL of potassium phosphate buffer (0.5 mM, pH 6.8) and 100 µL of L-DOPA (1 mM) and pre-incubated at 37 °C for 5 min in the dark. After the pre-incubation time, 30 µL of tyrosinase (100 U/mL) was added and the absorbance was measured at 475 nm, and every minute thereafter for 15 min, using the microplate reader. Kojic acid was used as the standard, and the results were expressed as a percentage of the control.

#### 4.3.3. Antimicrobial Activity

The antimicrobial activity of *S. polyschides* fractions was tested against three skin microorganisms, namely *Staphylococcus epidermidis* (DSM 1798), *Cutibacterium acnes* (DSM 1897), and *Malassezia furfur* (DSM 6170), previously obtained from the Leibniz Institute DSMZ-German Collection of Microorganisms and Cell Cultures (DSMZ) biobank. The effect on microbial growth of each fraction (200 µg/mL) was determined during the exponential phase growth, at 600 nm. Oxytetracycline was used as a positive control for *S. epidermidis* and *C. acnes,* and amphotericin B for *M. furfur.*

### 4.4. Inflammatory and Anti-Inflammatory Potential on RAW 264.7 Cells

#### 4.4.1. Cell Culture and Maintenance

Murine macrophages (RAW 264.7) (ATCC-TIB-71) were acquired from the American Type Culture Collection (ATCC) biobank. RAW 264.7 cells were grown in DMEM (Dulbecco’s Modified Eagle’s Medium) without phenol red, supplemented with 10% FBS, 1% antibiotic/antimycotic solution (Biowest, Riverside, MO, USA), and 1% sodium pyruvate (humidified atmosphere with 5% CO_2_ at 37 °C). Cells were seeded in 96-well microplates (5 × 10^4^ cells/well) for cell viability and nitric oxide (NO) production assessment. For determination of interleukins’ levels, the cells were seeded in 12-well microplates (5 × 10^5^ cells/well).

#### 4.4.2. Cell Viability and Nitric Oxide Production of RAW 264.7 Cells Induced by Lipopolysaccharide (LPS)

The cytotoxic effects of *S. polyschides* fractions were estimated using the 3-[4,5-dimethylthiazol-2-yl]-2,5-diphenyltetrazolium bromide (MTT) method [65]. RAW 264.7 cells were treated with a non-toxic concentration (20 µg/mL) of *S. polyschides* fractions for 24 h, and their inflammatory potential verified through NO level’s analysis. To determine the anti-inflammatory potential, RAW 264.7 cells were pre-treated with a non-toxic concentration (20 µg/mL) of *S. polyschides* fractions for 1 h and then subjected to an inflammatory condition induced by LPS at 1 µg/mL over 24 h. NO production was determined using the Griess reagent ((1% (*w*/*v*) sulfanilamide, 0.1% (*w*/*v*) *N*-(1-naphthyl) ethylenediamine in 2.5% (*v*/*v*) phosphoric acid)) as described by Yang et al. [66]. Dexamethasone (DEX) (20 µg/mL) was used as the positive control. Results were expressed as a percentage of the control (untreated cells).

#### 4.4.3. Pro-Inflammatory and Anti-Inflammatory Cytokines Production

RAW 264.7 cells were pre-incubated with a non-toxic concentration (20 µg/mL) of *S. polyschides* fractions for 1 h, and then exposed to LPS (1 µg/mL) for 18 h. The TNF-α, IL-6 and IL-10 cytokines levels (Thermoscientific, Vienna, Austria) were determined using ELISA kits (TNF-alpha mouse uncoated, IL-6 Ready-SET Go and IL-10 mouse uncoated, respectively) according to the suppliers’ instructions. TNF-α, IL-6 and IL-10 levels were expressed as a percentage of the control (untreated cells).

### 4.5. Photoprotective Capacity in 3T3 Cells

#### 4.5.1. Cell Culture and Maintenance

3T3 fibroblasts (ACC-173) were attained from the DSMZ biobank and cultivated according to supplier information. Cells were grown in DMEM F12 (Dulbecco’s Modified Eagle’s Medium: Nutrient Mix F12), supplemented with 10% fetal bovine serum (FBS), 100 IU/mL penicillin, and 100 µg/mL streptomycin in a humidified atmosphere with 5% CO_2_ at 37 °C. Subculture was performed according to biobank instructions whenever the cultures reached 80–85% of confluence. Cells were seeded in 96-well plates at a density of (5 × 10^4^ cells/well) and incubated until total confluence for experiments.

#### 4.5.2. Cell Viability and Reactive Oxygen Species (ROS) Production

The cytotoxic effects of *S. polyschides* fractions were estimated using the MTT colorimetric assay [65]. Saponin (Sigma-Aldrich, Steinheim, Germany) was used as a cellular death positive control. The results were expressed as a percentage of the control (untreated cells). The photoprotective capacity of *S. polyschides* fractions was determined by evaluating the ROS levels after exposure to UVR, as described by Marto et al. [67] with minor modifications [18]. Cells were treated with *S. polyschides* fractions at non-toxic concentrations (10 µg/mL) for 1 h, at 37 °C, in the dark. Treated cells were then exposed to UV radiation (12.5 mJ/cm^2^) for 1 h, in a UV curing chamber (UVA Cube 400, Hönle Technology, Gräfelfing, Germany). Later, 100 µL of 2′,7′-dichlorodihydrofluorescein diacetate (H2-DCFDA) (20 mM) was added to the cells, which were then incubated for 30 min, at 37 °C, in the dark. ROS levels were determined by measuring fluorescence (λ excitation: 495 nm; λ emission: 527 nm) every minute, for 10 min. *N*-acetyl-l-cysteine (NAC) (10 µg/mL) was used as the positive control. The results were expressed as a percentage of the control.

### 4.6. NMR Chemical Profile

The chemical profile of *S. polyschides* fractions (F1–F5) was attained by proton nuclear magnetic resonance (^1^H NMR) spectroscopy in a Bruker Avance spectrometer (Bruker, Madrid, Spain ) at 400.13 MHz. Spectra were obtained as described in Susano et al. [18]. The samples (c.a 5–6 mg) were dissolved in 0.5 mL of deuterated solvents (CDCl_3_, MeOD or D_2_O; Sigma-Aldrich, St. Louis, MO, USA) and the spectra were recorded at 25 °C. Chemical shifts (δ) were expressed in ppm and referenced to the residual solvent signal (δH = 7.26 ppm, CDCl_3_; δH = 3.31 ppm, MeOD; δH = 4.79 ppm, D_2_O).

### 4.7. Data and Statistical Analysis

Data were checked for normality and homoscedasticity using the Shapiro-Wilk and Levene’s test, respectively. The differences between the samples and controls were determined using one-way analysis of variance (ANOVA), with Dunnett’s multiple comparison tests, and the data that did not meet normal distribution were compared with the Kruskal-Wallis non-parametric test [68]. Differences were considered significant at a level of 0.05 (*p* < 0.05). All data were obtained from at least three independent experiments with four replicates, and the results are presented as mean ± standard error of the mean (SEM). The IC_50_/EC_50_ values were determined using the GraphPad v5.1 software (GraphPad Software, La Jolla, CA, USA) and the equation y = 100/(1 + 10 ^(X − LogIC^_50_^)^).

## 5. Conclusions

The diethyl ether (F2) and ethyl acetate (F3) fractions from *S. polyschides* demonstrated relevant bioactive properties (antioxidant, anti-enzymatic, antimicrobial, anti-inflammatory and photoprotective), thus showing potential to be incorporated into skincare formulations. In addition, the crude extract (F1) and water-insoluble fraction (F5) also revealed capacity for application in new skincare products since they demonstrated antimicrobial and anti-inflammatory properties. More detailed chemical studies of each fraction by complementary analytical techniques, such as high-performance liquid chromatography coupled to tandem mass spectrometry (LC-MS/MS^n^), and/or isolation and structural characterization of bioactive compounds, will be the next step to complement the ^1^H NMR screening here reported. Nevertheless, this work demonstrates for the first time the dermatological potential of the seaweed *S. polyschides*, highlighting its relevance to be further investigated as a source of natural active ingredients for novel, sustainable, and effective topical skincare formulations.

## Figures and Tables

**Table 1:** Antioxidant capacity of *Saccorhiza polyschides*.

Fraction	TPC ^a^	DPPH ^b^	FRAP ^c^	ORAC ^d^
**F1**	5.8 ± 0.2	>200	44.5 ± 4.7	221.6 ± 17.4
**F2**	95.5 ± 9.0	>200	1081.6 ± 33.3	1715.8 ± 44.6
**F3**	199.9 ± 23.7	>200	828.2 ± 21.2	3126.0 ± 225.1
**F4**	2.2 ± 0.5	>200	0.6 ± 0.1	26.5 ± 0.7
**F5**	19.1 ± 1.3	>200	214.1 ± 16.1	471.4 ± 17.6
**BHT**	-	164.5 (142.7–189.7)	2821.5 ± 51.5	142.9 ± 9.1

^a^ mg of phloroglucinol equivalents/g extract (mg PE/g); ^b^ radical scavenging activity (EC_50_ µg/mL); ^c^ µM of FeSO_4_ equivalents/g extract (µM FeSO_4_/g); ^d^ µmol of Trolox equivalents/g extract (µmol TE/g). EC_50_ values were determined for a 95% confidence interval. BHT (3,5-di-*ter*t-4-butylhydroxytoluene).

**Table 2:** Antimicrobial activity (IC_50_, µg/mL) of *Saccorhiza polyschides* fractions, oxytetracycline and amphotericin B.

Fraction	*Cutibacterium acnes*	*Staphylococcus epidermidis*	*Malassezia furfur*
**F1**	39.0 (26.9–52.1)	>200	>200
**F2**	33.4 (23.6–46.9)	>200	>200
**F3**	>200	>200	>200
**F4**	>200	>200	>200
**F5**	12.4 (6.6–17.6)	>200	>200
**Oxytetracycline**	0.07 (0.05–0.09)	12.4 (11.2–16.1)	-
**Amphotericin B**	-	-	11.4 (8.6–15.0)

## Data Availability

The data presented in this study are available upon request from the corresponding author.

## Sample Availability

Samples of the studied fractions are available from the authors.
